# Associations Between MRI Findings and Pain, Joint Sounds, and Limited Mouth Opening in Patients with Temporomandibular Disorders

**DOI:** 10.3390/jcm15218204

**Published:** 2026-10-22

**Authors:** Gonca Sağlam Akkaya, Selçuk Akkaya

**Affiliations:** 1 Department of Physical Medicine and Rehabilitation, Faculty of Medicine, Karadeniz Technical University, 61080 Trabzon, Türkiye; 2 Department of Radiology, Faculty of Medicine, Karadeniz Technical University, 61080 Trabzon, Türkiye; selcuk.akkaya85@gmail.com

**Keywords:** temporomandibular joint disorders, magnetic resonance imaging, disc displacement

## Abstract

**Background/Objectives:** Associations between temporomandibular joint (TMJ) magnetic resonance imaging (MRI) findings and clinical manifestations vary across studies. This study aimed to examine associations of disc position and MRI findings with joint pain and sounds and to explore their relationships with limited mouth opening. **Methods:** This retrospective cross-sectional study included 108 patients and 216 TMJs. MRI assessment included disc position, disc morphology, condylar changes, and effusion volume. Analyses of pain, clicking, and crepitus accounted for within-patient correlation between bilateral joints. Maximum assisted mouth opening was measured, and limited mouth opening was analyzed separately for each patient. **Results:** Anterior disc displacement with reduction (ADDwR) and anterior disc displacement without reduction (ADDwoR) showed nominal associations with joint pain (adjusted odds ratio [OR] 3.60, 95% confidence interval [CI] 1.89–6.86; OR 2.90, 95% CI 1.14–7.36, respectively) and clicking (OR 5.70, 95% CI 2.56–12.70; OR 3.71, 95% CI 1.15–11.93, respectively), compared with normal disc position. Condylar erosion was not associated with pain (OR 2.62, 95% CI 0.92–7.50). ADDwoR and condylar erosion in at least one joint were associated with limited mouth opening (OR 3.61, 95% CI 1.43–9.11; OR 2.98, 95% CI 1.19–7.45, respectively). After adjustment for multiple comparisons, the associations of ADDwR with pain and clicking remained statistically significant (both q = 0.004), whereas those of ADDwoR did not reach statistical significance (both q = 0.053). **Conclusions:** ADDwR was associated with joint pain and clicking, whereas ADDwoR and condylar erosion in at least one joint were associated with limited mouth opening. However, these findings should be interpreted in light of the retrospective design, the absence of asymptomatic controls, and nominal significance thresholds.

## 1. Introduction

The temporomandibular joint (TMJ) is a complex synovial articulation comprising an articular disc, mandibular condyle, articular surfaces, and supporting soft tissues that coordinate functional mandibular movements [1,2]. Temporomandibular disorders (TMD) comprise intra-articular and masticatory muscle conditions and are influenced by psychosocial factors. They may present with regional pain, joint sounds, or restricted jaw opening; these manifestations do not necessarily identify a specific intra-articular lesion [3,4,5].

Magnetic resonance imaging (MRI) depicts the articular disc and associated soft tissues and can contribute to the assessment of intra-articular TMJ conditions [6]. Although MRI is commonly used to evaluate structural and positional variations, its findings should be interpreted alongside clinical symptoms.

Imaging findings may not establish the cause of pain or other symptoms. Prior studies have examined associations between MRI findings and clinical symptoms, with variable results [7,8,9]. Differences in referral populations, clinical outcome definitions, imaging protocols, and assessment methods may contribute to this variation. The recent literature highlights variable associations between imaging findings and clinical symptoms [10,11]. 

This study aimed to evaluate the relationship of disc position and MRI parameters including disc morphology, condylar changes, and effusion volume with joint pain and joint sounds, adjusting for within-patient correlation between bilateral TMJs. Exploratory aims focused on analyzing patient-level restricted mouth opening and comparing imaging profiles among disc-position categories.

## 2. Materials and Methods

### 2.1. Study Population

This single-center, retrospective cross-sectional study was conducted at the Departments of Physical Medicine and Rehabilitation and Radiology, Karadeniz Technical University Farabi Hospital, a tertiary referral center. Electronic clinical and radiological records of consecutive patients evaluated for TMD from January 2020 through July 2024 were reviewed, after obtaining ethical approval (dated 31 July 2024, protocol: 2024/140). Eligible patients were adults with persistent TMJ pain, clicking, crepitus, and/or limited mouth opening who underwent diagnostic MRI after an inadequate response to initial conservative management, including patient education, a soft diet, and nonsteroidal anti-inflammatory drugs.

Exclusion criteria included incomplete clinical records or MRI examinations of inadequate diagnostic quality, including those affected by artifacts; occlusal splint therapy, intra-articular TMJ injections, or TMJ surgery within the six months preceding the clinical examination; systemic inflammatory or rheumatological joint disease; and maxillofacial trauma, condylar fractures, ankylosis, tumors, or malformations.

Each patient contributed one paired clinical and MRI assessment. For patients with repeated examinations, the first eligible assessment before subsequent treatment was retained.

### 2.2. Clinical Assessment

Demographic data, symptom histories, TMJ pain, joint sounds (clicking and crepitus), and maximum mouth opening (MMO) were retrospectively extracted from medical records. All clinical assessments were based on the Diagnostic Criteria for Temporomandibular Disorders (DC/TMD) [12] and were performed and documented by a specialist in Physical Medicine and Rehabilitation.

Pain was defined as pain localized to the preauricular region or TMJ area and documented at the baseline assessment as spontaneous pain, pain during jaw function, or pain elicited by standardized digital palpation and functional mandibular movements. Clicking and crepitus were assessed separately for the right and left joints using manual palpation and auditory detection during mandibular opening and closing. Each finding was attributed to the corresponding joint.

MMO was measured in millimeters using a calibrated sliding caliper (Insize Co., Ltd., Suzhou, China)as the maximum assisted interincisal distance between the maxillary and mandibular central incisors during examiner-applied passive stretch, adding vertical incisal overlap where applicable. The limited mouth opening (LMO) classification documented in the clinical records was retained as the primary functional outcome. For the separate sensitivity analysis, restricted mouth opening was defined as assisted MMO < 40 mm, corresponding to the opening criterion for disc displacement without reduction with limited opening in DC/TMD [12].

### 2.3. Radiological Evaluation

Bilateral TMJ MRI examinations were performed using a 3 T scanner (Magnetom Skyra, Siemens Healthineers, Erlangen, Germany) equipped with a dedicated TMJ surface coil. The imaging protocol included sagittal oblique T2-weighted and proton-density (PD) fast spin-echo sequences in closed- and open-mouth positions and coronal oblique T1-weighted sequences in the closed-mouth position. Section thickness was 3 mm.

A radiologist with more than 10 years of experience in temporomandibular imaging assessed the examinations while blinded to clinical findings, symptom history, and patient identifiers. For intrareader assessment, the same radiologist reread a random subset of 30 patients (60 bilateral TMJs) after an eight-week interval, blinded to initial ratings, clinical data, and patient identities. Categorical agreement was evaluated using Cohen’s kappa (κ); quantitative agreement used two-way random-effects intraclass correlation coefficients (ICCs) with absolute agreement for single measures.

Disc position was classified on sagittal oblique images as normal, anterior disc displacement with reduction (ADDwR), or anterior disc displacement without reduction (ADDwoR). Anterior displacement was identified when the junction of the posterior band and bilaminar zone lay anterior to the 11 o’clock position relative to the condylar head on closed-mouth images. Reduction was defined by restoration of the normal condyle–disc relationship on open-mouth images [4].

**Figure 1:**
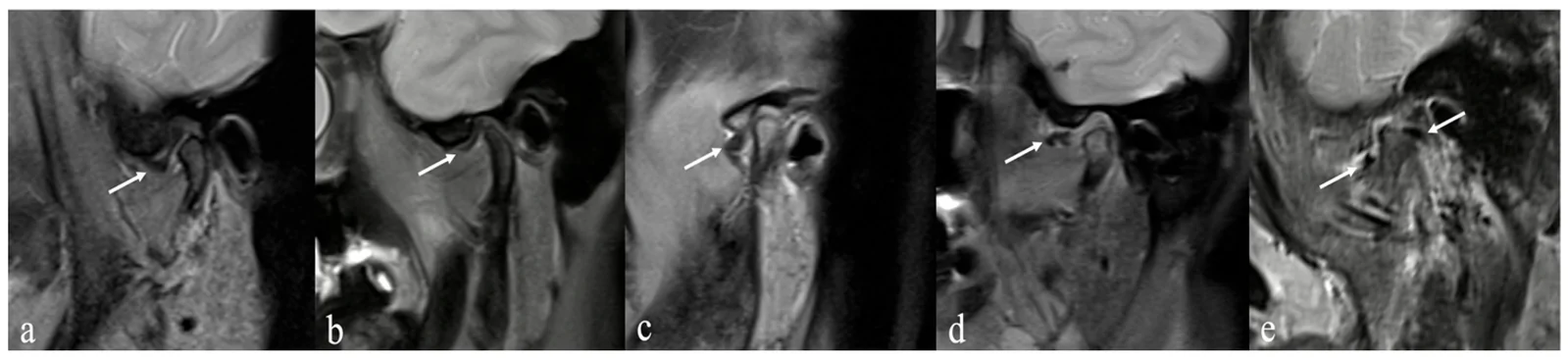
Disc configurations (arrows) on proton-density (**a**–**d**) and T2-weighted (**e**) images. (**a**) Biconcave disc; (**b**) flattened disc; (**c**) rounded disc; (**d**) crumpled disc; (**e**) perforated disc.

Disc configuration was assessed in the closed-mouth position and classified as biconcave (distinct anterior and posterior bands with a thin intermediate zone), flattened (relatively uniform thickness), rounded (convex superior and inferior borders), crumpled (folded or irregular borders), or perforated [13,14]. MRI-suspected perforation was defined as a structural discontinuity, full-thickness defect, or complete disruption of the intermediate zone on sagittal oblique T2-weighted and PD images, with apparent fluid-signal or soft-tissue continuity between the superior and inferior joint compartments. The perforation category represents an MRI classification (Figure 1).

**Figure 2:**
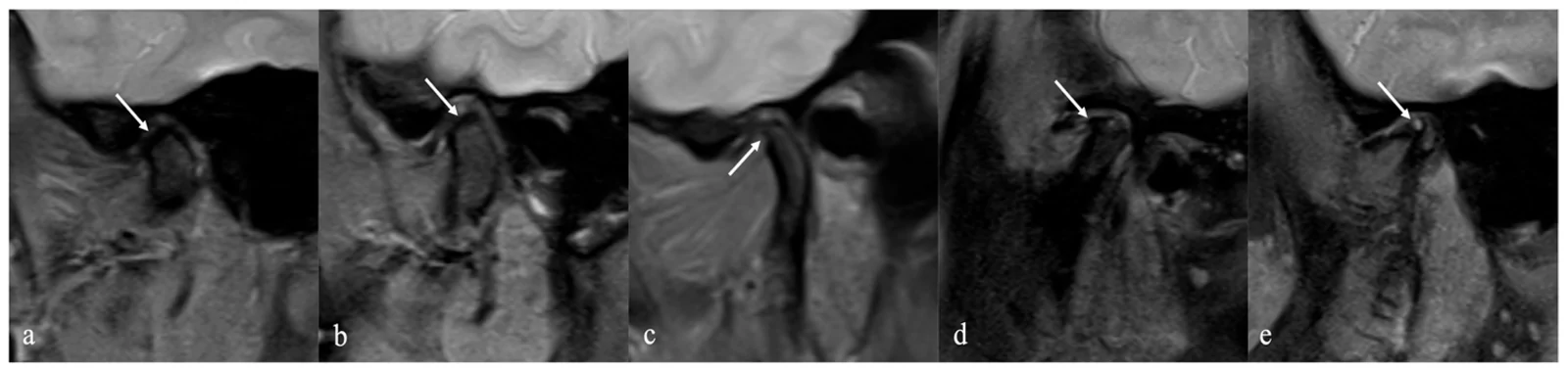
Condylar degenerative changes (arrows) on proton-density images. (**a**) Flattening; (**b**) sclerosis; (**c**) erosion; (**d**) osteophyte; (**e**) subchondral cyst.

Condylar changes were classified as flattening (loss of the rounded condylar contour), erosion (a cortical defect), osteophyte (a marginal osseous prominence), subchondral cyst (a subarticular cavity), or sclerosis [15,16]. MRI features interpreted as sclerosis comprised well-defined areas of pronounced low signal on T1-weighted and PD/T2-weighted sequences involving the subchondral cortex and adjacent trabecular bone, with apparent cortical thickening or compaction (Figure 2). Degeneration status was summarized as no recorded finding, one finding, or two or more findings.

Equivocal findings were reassessed by cross-referencing sagittal and coronal views and the available sequences. Borderline contour changes, signal abnormalities, and disc positions were interpreted conservatively against the predefined morphological criteria, with the final classification based on concordance across planes and sequences.

TMJ effusion volumetry was performed using a sagittal oblique proton density-weighted (PD) sequence acquired with a slice thickness of 3 mm and no interslice gap. Volumetric measurements were conducted using dedicated medical imaging software (syngo.via VB40B, Siemens Healthineers, Erlangen, Germany).

The assessment encompassed both the superior and inferior joint spaces of the TMJ. Delineation rules required manual region-of-interest (ROI) contouring along the hyperintense synovial fluid boundaries within the synovial cavity on each consecutive slice. To minimize partial-volume effects, multiplanar reformations (MPR) were used for anatomical cross-referencing, and mixed-signal border voxels at the fluid–bone and fluid–soft tissue interfaces were excluded from the ROI contours.

**Figure 3:**
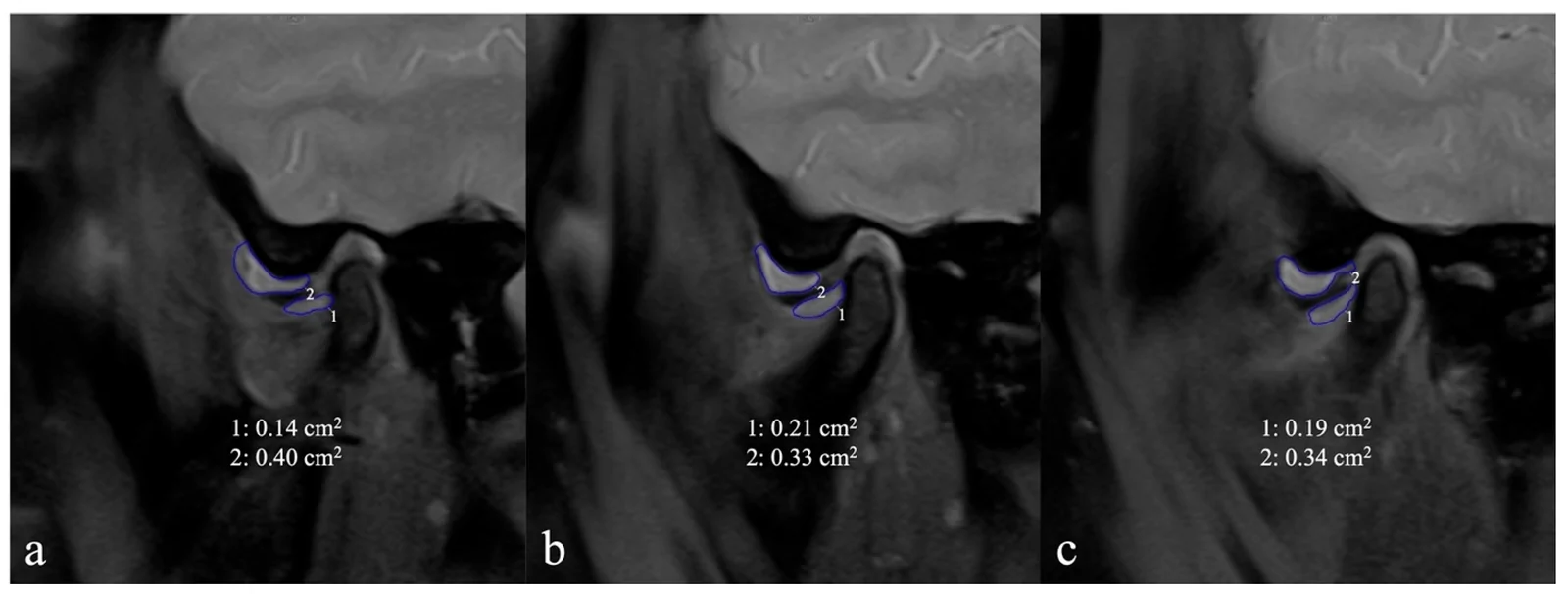
Temporomandibular joint effusion on consecutive sagittal oblique proton-density-weighted images: (**a**) first slice; (**b**) second slice; (**c**) third slice. Blue contours outline effusion in the inferior (1) and superior (2) joint spaces.

The volume of joint effusion was estimated using a volumetric method, whereby the effusion area on each slice was multiplied by the corresponding slice thickness, with the resulting slice volumes summed to yield the total volume (Figure 3). Outlined areas displayed in cm^2^ were converted to mm^2^ before multiplication, and volumes were expressed in mm^3^. The hip-fluid study provides a methodological precedent for fluid quantification [17].

The measurement protocol, descriptive findings, and corresponding MRI illustration for inferior lateral pterygoid muscle tendon thickness are provided in Supplementary Material S1 (Table S1 and Figure S1).

### 2.4. Statistical Analysis

Before data collection, sample size and power were calculated using G*Power 3.1.9.7 (Heinrich Heine University Düsseldorf, Düsseldorf, Germany) and cluster-adjusted logistic regression formulas (two-sided α = 0.05; target power = 80%; baseline pain and LMO event rates = 50%). For bilateral joints, m = 2 and an assumed ICC of 0.30 gave a design effect of 1 + (m − 1)ICC = 1.30. Under these assumptions, 108 patients (216 joints) provided sufficient power to detect odds ratios (ORs) ≥ 2.50 for primary joint-level predictors and ≥3.00 for patient-level LMO models.

Statistical analyses were performed using IBM SPSS Statistics (version 23.0, IBM Corp., Armonk, NY, USA) and Python (version 3.10, statsmodels package version 0.14.0; Python Software Foundation, Wilmington, DE, USA). Categorical variables are expressed as frequencies and percentages, and continuous variables as mean ± standard deviation (SD) or median with interquartile range (IQR), according to their distributions. Associations were reported as adjusted ORs with 95% confidence intervals (CIs).

Joint-level binary outcomes (pain, clicking, and crepitus) were analyzed using population-averaged logistic generalized estimating equations (GEE) with a binomial distribution and logit link. Patient identifier defined the 108 clusters, each comprising two joints. An exchangeable working correlation structure and robust sandwich standard errors accounted for bilateral within-patient correlation. All models were adjusted for age (per 10-year increase, centered at 50 years) and sex.

Patient-level LMO was analyzed using multivariable logistic regression in 108 patients, retaining the clinical-record classification as the primary outcome (52 events). Assisted MMO < 40 mm was analyzed separately as a sensitivity outcome (52 events). Bilateral imaging findings were summarized by the presence of ADDwoR or condylar erosion in either joint. Both models included age and sex, with four nonintercept coefficients; the primary LMO model therefore had 13 events per coefficient.

Covariates were selected according to clinical relevance and outcome frequency, without automated selection. The pain model included disc position, erosion, age, sex, and log(1 + effusion volume in mm^3^). The clicking model included disc position, disc configuration (biconcave, flattened, or other/deformed), age, and sex; rounded, crumpled, and perforated discs were pooled in the other/deformed category. The crepitus model included disc position, condylar flattening, age, and sex. A separate patient-level osseous LMO model included flattening, erosion, subchondral cysts, age, and sex, with each MRI feature defined by its presence in either joint. Normal disc position and biconcave configuration were the reference categories.

Pain and clicking models contained six nonintercept coefficients, and crepitus and the additional osseous LMO model contained five. Joint-level event counts were 138 for pain, 63 for clicking, and 28 for crepitus. Modeled variables were complete after eligibility screening, and no imputation was used. All models converged without detected separation; maximum variance inflation factors (VIFs) ranged from 1.25 to 2.77.

Two-sided *p* < 0.05 was used as the nominal significance threshold. For multiple comparisons, Benjamini–Hochberg (BH) false discovery rate correction was applied across the 15 primary MRI predictor tests in the pain, clicking, crepitus, recorded LMO, and assisted MMO < 40 mm sensitivity models. BH-adjusted q values are reported alongside nominal *p* values.

## 3. Results

### 3.1. Study Population and Clinical Characteristics

Of 142 patients screened, 34 were excluded due to the incomplete clinical records or diagnostically inadequate MRI quality, including artifacts (*n* = 12); occlusal splint therapy, intra-articular TMJ injections, or TMJ surgery within the six months preceding the clinical examination (*n* = 8); systemic inflammatory or rheumatological joint disease (*n* = 7); or maxillofacial trauma, condylar fractures, ankylosis, tumors, or malformations (*n* = 7). The overall exclusion rate was 23.9%. The final analytical cohort comprised 108 patients with complete clinical and bilateral MRI data, yielding 216 joints. The interval between clinical examination and MRI was approximately one month (mean ± standard deviation, 30.2 ± 6.4 days; range, 14–45 days).

The mean patient age was 49.5 ± 14.5 years and 91/108 (84.3%) were female. At the joint level, pain was recorded for 138/216 (63.9%), clicking for 63/216 (29.2%), and crepitus for 28/216 (13.0%). At the patient level, LMO was recorded for 52/108 (48.1%) (Table 1).

### 3.2. MRI Findings

Among 216 joints, flattened disc configuration was recorded in 113 (52.3%), biconcave in 55 (25.5%), rounded in 23 (10.6%), crumpled in 21 (9.7%), and perforated in 4 (1.9%). Disc position was normal in 80 joints (37.0%), ADDwR in 79 (36.6%), and ADDwoR in 57 (26.4%).

Condylar flattening was recorded in 127 (58.8%), sclerosis in 75 (34.7%), osteophytes in 73 (33.8%), erosion in 45 (20.8%), and subchondral cysts in 21 (9.7%). No recorded degenerative feature was present in 61 joints (28.2%), one was present in 51 (23.6%), and there were multiple features in 104 (48.1%).

Mean patient-level assisted MMO was 39.1 ± 5.8 mm in the 108 bilateral records. Mean joint-level effusion volume was 61.7 ± 142.8 mm^3^ in 216 joint records. Effusion volume was markedly asymmetric, with a pooled median of 0 mm^3^ (IQR 0–61.5 mm^3^) (Table 2). Table 3 reports medians and IQRs by disc-position group.

Incidental MRI findings among 216 joints included 10 recorded instances of mastoiditis (4.6%), four maxillary sinus retention cysts (1.9%), two synovial cysts (0.9%), and one each of cervical lymphadenopathy and branchial cleft cyst (0.5%).

In the repeat-reading subset of 30 patients (60 bilateral TMJs), Cohen’s κ values (95% CIs) were 0.88 (0.79–0.96) for disc position, 0.85 (0.75–0.95) for disc configuration, 0.90 (0.82–0.98) for condylar flattening, 0.86 (0.76–0.96) for condylar erosion, 0.89 (0.80–0.97) for condylar sclerosis, 0.91 (0.83–0.98) for osteophytes, and 0.84 (0.74–0.94) for subchondral cysts. The absolute-agreement ICC for single measures was 0.92 (95% CI 0.85–0.96) for effusion volume.

### 3.3. Associations Between Disc Position and Clinical/MRI Variables

Pain occurred in 38/80 joints with normal disc position, 58/79 with ADDwR, and 42/57 with ADDwoR. Clicking occurred in 13/80, 36/79, and 14/57, respectively; crepitus in 5/80, 9/79, and 14/57. Biconcave discs predominated in normal-position joints (47/80), flattened discs in ADDwR (57/79), and rounded, crumpled, and perforated discs in ADDwoR (17/57, 13/57, and 4/57). Effusion-volume medians were 0, 38, and 72 mm^3^, respectively. These are descriptive joint-group comparisons.

In clustered joint-level analysis, relative to normal disc position, ADDwR was associated with pain (adjusted OR 3.60, 95% CI 1.89–6.86; *p* < 0.001; BH-adjusted q = 0.004) and ADDwoR with pain (2.90, 1.14–7.36; *p* = 0.025; q = 0.053). Erosion had an imprecise estimate (2.62, 0.92–7.50; *p* = 0.073; q = 0.110). In the patient-level analysis, ADDwoR in either joint (3.61, 1.43–9.11; *p* = 0.006; q = 0.030) and erosion in either joint (2.98, 1.19–7.45; *p* = 0.020; q = 0.050) were associated with the recorded LMO outcome. The separate sensitivity outcome of assisted MMO < 40 mm yielded 52/108 events and comparable associations for any ADDwoR (3.00, 1.20–7.51; *p* = 0.019; q = 0.050) and any erosion (3.32, 1.32–8.34; *p* = 0.011; q = 0.041) (Table 4).

In the clustered clicking model, ADDwR (adjusted OR 5.70, 95% CI 2.56–12.70; *p* < 0.001; q = 0.004) and ADDwoR (3.71, 1.15–11.93; *p* = 0.028; q = 0.053) were associated with clicking relative to normal disc position. In the clustered crepitus model, the ADDwoR estimate was 2.38 (1.00–5.67; nominal *p* = 0.050; BH-adjusted q = 0.083), but only 28 joints had crepitus and this borderline association should be considered exploratory. The collapsed disc-morphology terms in the clicking model were not independently associated with clicking. Table 5 provides the complete adjustment sets.

## 4. Discussion

TMJ pain, joint sounds, and restricted opening may arise from different articular and extra-articular processes, contributing to variable correspondence between clinical findings and MRI abnormalities [5,11]. In the present study, at nominal significance, disc displacement was associated with pain and clicking after accounting for bilateral joint dependence. At the patient level, LMO was related to ADDwoR and condylar erosion in either joint, while effusion volume varied descriptively across disc-position groups.

The association between disc displacement and pain is consistent with Matsubara et al., who linked ADDwoR to TMJ pain [7], and with Sancar et al., who found associations of both ADDwR and ADDwoR with pain [10]. The present estimates do not demonstrate that ADDwoR represents a greater pain association than ADDwR. The adjusted erosion estimate was imprecise and its confidence interval included unity. In contrast, Ohlmann et al. reported no significant relationship between MRI-defined disc displacement and TMJ arthralgia, in contrast to the disc–pain association observed in the present study. Effusion and osteoarthrosis were also not significantly related to arthralgia, whereas tenderness at the masseter origin and psychosocial factors were associated with TMJ pain [18]. Differences in clinical pain definitions and the assessment of myogenous and psychosocial factors could contribute to this discrepancy. Sang et al. identified pain associations with displacement, effusion, marrow edema, and erosion across a heterogeneous literature [11]. These findings support combined imaging and clinical assessment rather than attributing pain to a single MRI feature [8,9].

Disc displacement does not consistently result in pain. Larheim et al. identified disc displacement in 35% of asymptomatic volunteers, although the extent and reduction behavior differed from those observed in symptomatic patients [19]. Similarly, Katzberg and colleagues reported disc abnormalities in asymptomatic individuals [13,20]. A plausible mechanism linking disc displacement to arthralgia involves altered load distribution and persistent stress on the vascularized and innervated retrodiscal tissues, particularly in ADDwoR. Compression and shear forces can lead to microtrauma, impaired local perfusion, and synovial irritation, with cytokines and prostaglandins contributing to nociception. Tissue adaptation may explain the absence of pain in some individuals despite the presence of disc displacement [2,3,14].

Clicking occurred most often in ADDwR, consistent with disc recapture during jaw movement. Associations of both displacement types with clicking resemble Jeon et al.’s findings for TMJ sounds [21], although their outcome combined joint sounds. Topaloglu Yasan et al. reported no significant associations between MRI findings and joint noise [9]. Differences in outcome definitions and populations may contribute to this discrepancy. The ADDwoR–clicking association does not demonstrate disc reduction on MRI; intermittent mechanical behavior and the examination–MRI interval may explain disagreement.

The anatomical study of Widmalm et al. and the clinical radiographic study by Arayasantiparb et al. support relationships between joint sounds, altered joint structure, and degenerative changes [22,23]. In the present study, crepitus occurred in all disc-position categories. The ADDwoR estimate was borderline, and condylar flattening was not independently associated with crepitus in the adjusted model. Differences in cartilage or surface abnormalities, sound detection, and the distinction between remodeling and destructive changes may contribute to the variation between studies.

The association of ADDwoR in either joint with LMO agrees with Topaloglu Yasan et al., who also reported an independent relationship between nonreducing displacement and restricted opening [9]. Studies comparing clinical and MRI diagnoses show that clinical findings do not map perfectly to a specific displacement subtype [24,25]. A persistently displaced disc may impede condylar translation, but restriction can also reflect pain-related guarding, muscular dysfunction, or adhesions [14,26].

Biconcave discs predominated in joints with normal disc position, flattened discs in ADDwR, and rounded or crumpled discs in ADDwoR. Matsubara et al. reported comparable relationships between normal configuration and normal disc position, flattening and ADDwR, and folding and ADDwoR [7]. Similarly, another study demonstrated that deformed configurations were more frequently linked with ADDwoR [9]. In the present study, however, disc morphology was not independently associated with clicking after multivariable adjustment.

Condylar abnormalities were more frequent in ADDwoR, with multiple findings in 51/57 joints. This coexistence agrees with studies linking internal derangement and osseous changes [2,15,21], although flattening and sclerosis may reflect adaptation as well as degeneration. Erosion in either joint was associated with LMO, but the wide confidence intervals for flattening and subchondral cysts require caution. Palconet et al. found poor correlations of osseous severity with pain and mandibular range of motion [27].

Effusion-volume medians were higher in the displaced-disc categories, supporting the coexistence of fluid accumulation and internal derangement described in earlier studies [7,28]. However, measured volume was not independently associated with pain in the adjusted model of the present study. This differs from the associations of high-grade effusion with pain reported by Matsubara et al. and Jeon et al. [7,21], and resembles Sancar et al.’s finding of no clear effusion–symptom relationship [10]. Measurable effusion was absent in at least half of the joints, while a few high-volume observations produced a markedly right-skewed distribution. The log(1 + volume) transformation compressed the upper tail but did not alter the concentration of observations at zero. This pattern may have limited the information available for estimating a graded relationship between effusion volume and the odds of joint pain, potentially contributing to the nonsignificant association in the adjusted model.

Strengths of this study include bilateral MRI evaluation, blinded image interpretation, assessment of intrareader repeatability, and analyses accounting for within-patient joint correlation. However, the retrospective, single-center design and absence of an asymptomatic control group limit generalizability. Psychosocial and myogenous variables were not included in the analyses, leaving potential confounding by these factors unaddressed. The primary LMO outcome was based on clinical records rather than the standardized measurement protocol, which may introduce misclassification bias; however, the sensitivity analysis using assisted MMO < 40 mm yielded comparable results, which provides some reassurance. The approximately one-month interval between clinical examination and MRI could have allowed changes in symptoms or joint mechanics. The sample size, infrequent outcomes, correlated imaging findings, and multiple testing also limit interpretation. Manual delineation and partial-volume effects introduce measurement uncertainty despite intrareader repeatability, and interreader agreement was not assessed. The cross-sectional design cannot establish causality or progression. Prospective studies incorporating standardized clinical assessment, psychosocial and myogenous variables, opening-path measurements, and reproducible quantitative MRI should evaluate the clinical usefulness of these findings.

## 5. Conclusions

TMJ MRI findings should be interpreted alongside clinical symptoms and measured mandibular function. ADDwR was associated with pain and clicking after multiple-comparison correction, whereas the corresponding ADDwoR associations were borderline. ADDwoR and condylar erosion in either joint were associated with LMO. The clinical significance and potential utility of these associations require confirmation in prospective studies.

## Figures and Tables

**Table 1:** Patient characteristics (*n* = 108) and recorded joint-level symptoms (*n* = 216 joints).

Measure	Value	Unit/Denominator
Age, mean ± SD	49.5 ± 14.5	years; 108 patients
Female	91 (84.3%)	108 patients
Male	17 (15.7%)	108 patients
Pain	138 (63.9%)	216 joints
Clicking	63 (29.2%)	216 joints
Crepitus	28 (13.0%)	216 joints
Limited mouth opening	52 (48.1%)	108 patients

SD, standard deviation. Denominators are shown in the final column.

**Table 2:** Descriptive MRI findings in 216 joint records and maximum assisted mouth opening in 108 bilateral records.

Domain	Finding	n (%) or Summary Statistic
Disc configuration	Biconcave	55 (25.5%)
	Flattened	113 (52.3%)
	Rounded	23 (10.6%)
	Crumpled	21 (9.7%)
	MRI-suspected perforation	4 (1.9%)
Disc position	Normal	80 (37.0%)
	ADDwR	79 (36.6%)
	ADDwoR	57 (26.4%)
Condylar changes	Flattening	127 (58.8%)
	Sclerosis	75 (34.7%)
	Erosion	45 (20.8%)
	Osteophytes	73 (33.8%)
	Subchondral cyst	21 (9.7%)
Condylar degeneration status	No recorded finding	61 (28.2%)
	One finding	51 (23.6%)
	Multiple findings	104 (48.1%)
Incidental findings	Branchial cleft cyst	1 (0.5%)
	Mastoiditis	10 (4.6%)
	Maxillary sinus retention cyst	4 (1.9%)
	Cervical lymphadenopathy	1 (0.5%)
	Synovial cyst	2 (0.9%)
Continuous measures	Maximum assisted mouth opening (mm; 108 patients)	39.1 ± 5.8
	Effusion volume, mean ± SD (mm^3^; 216 joints)	61.7 ± 142.8
	Effusion volume, median (IQR) (mm^3^; 216 joints)	0 (0–61.5)

Categorical MRI values are n (% of 216 joints). Continuous values are mean ± SD except the additional effusion median (IQR); measurement units and denominators are shown. ADDwR, anterior disc displacement with reduction; ADDwoR, anterior disc displacement without reduction.

**Table 3:** Descriptive clinical and MRI findings by disc position.

Finding	Normal (n = 80)	ADDwR (n = 79)	ADDwoR (n = 57)
Pain	38 (47.5%)	58 (73.4%)	42 (73.7%)
Click	13 (16.3%)	36 (45.6%)	14 (24.6%)
Crepitus	5 (6.3%)	9 (11.4%)	14 (24.6%)
Disc configuration—Biconcave	47 (58.8%)	8 (10.1%)	0 (0%)
Disc configuration—Flattened	33 (41.2%)	57 (72.2%)	23 (40.4%)
Disc configuration—Rounded	0 (0%)	6 (7.6%)	17 (29.8%)
Disc configuration—Crumpled	0 (0%)	8 (10.1%)	13 (22.8%)
Disc configuration—MRI-suspected perforation	0 (0%)	0 (0%)	4 (7%)
Condylar degenerative changes—Flattening	24 (30%)	52 (65.8%)	51 (89.5%)
Condylar degenerative changes—Sclerosis	19 (23.8%)	28 (35.4%)	28 (49.1%)
Condylar degenerative changes—Erosion	5 (6.3%)	12 (15.2%)	28 (49.1%)
Condylar degenerative changes—Osteophytes	3 (3.8%)	19 (24.1%)	51 (89.5%)
Condylar degenerative changes—Subchondral cyst	3 (3.8%)	2 (2.5%)	16 (28.1%)
Condylar degeneration status—Negative	45 (56.3%)	16 (20.3%)	0 (0%)
Condylar degeneration status—Single finding	19 (23.8%)	26 (32.9%)	6 (10.5%)
Condylar degeneration status—Multiple findings	16 (20%)	37 (46.8%)	51 (89.5%)
Effusion volume (mm3) [median (IQR)]	0 (0–0)	38 (0–75)	72 (0–189)

Values are n (%) of joints or median (IQR), as indicated. Columns contain 80 normal-position, 79 ADDwR, and 57 ADDwoR joints. Patient-level age, sex, MMO, and LMO are reported separately and are not duplicated in this joint-defined comparison.

**Table 4:** Cluster-adjusted pain model and patient-level limited mouth opening models.

Outcome/Model	Covariate	Adjusted OR (95% CI)	*p*	BH-Adjusted q
Pain GEE	ADDwR vs. normal	3.60 (1.89–6.86)	<0.001	0.004
	ADDwoR vs. normal	2.90 (1.14–7.36)	0.025	0.053
	Erosion	2.62 (0.92–7.50)	0.073	0.110
	Age, per 10 years	1.09 (0.86–1.39)	0.479	—
	Female vs. male	0.35 (0.14–0.90)	0.028	—
	log(1 + effusion volume)	1.11 (0.97–1.27)	0.146	0.183
Recorded LMO, patient level	Any ADDwoR	3.61 (1.43–9.11)	0.006	0.030
	Any erosion	2.98 (1.19–7.45)	0.020	0.050
	Age, per 10 years	1.08 (0.81–1.45)	0.589	—
	Female vs. male	0.73 (0.22–2.45)	0.614	—
Assisted MMO < 40 mm, sensitivity	Any ADDwoR	3.00 (1.20–7.51)	0.019	0.050
	Any erosion	3.32 (1.32–8.34)	0.011	0.041
	Age, per 10 years	1.05 (0.79–1.41)	0.734	—
	Female vs. male	0.85 (0.25–2.84)	0.792	—
Exploratory osseous LMO	Any condylar flattening	11.73 (2.87–47.91)	<0.001	Exploratory
	Any erosion	2.68 (0.90–7.95)	0.076	Exploratory
	Any subchondral cyst	13.04 (2.02–84.36)	0.007	Exploratory
	Age, per 10 years	1.08 (0.78–1.51)	0.643	—
	Female vs. male	0.84 (0.21–3.42)	0.813	—

OR, odds ratio; CI, confidence interval; ADDwR, anterior disc displacement with reduction; ADDwoR, anterior disc displacement without reduction. The pain GEE includes 216 joints, 138 pain events, and 108 patient clusters; its estimates are population-averaged and use robust standard errors. Recorded LMO includes 52 events in 108 patients; the assisted MMO < 40 mm sensitivity outcome includes 52. The patient-level predictors indicate a finding in either joint. Age is per 10-year increase, centered at 50 years; sex is female versus male; effusion is log(1 + volume in mm^3^). All displayed covariates were fitted simultaneously within each model. CIs are unadjusted; q values are Benjamini–Hochberg-adjusted across the 15 primary MRI predictor tests. The exploratory osseous LMO model was not included in this correction and is reported as exploratory.

**Table 5:** Cluster-adjusted models for clicking and crepitus.

Outcome/Model	Covariate	Adjusted OR (95% CI)	*p*	BH-Adjusted q
Clicking GEE	ADDwR vs. normal	5.70 (2.56–12.70)	<0.001	0.004
	ADDwoR vs. normal	3.71 (1.15–11.93)	0.028	0.053
	Flattened vs. biconcave	0.71 (0.25–2.02)	0.517	0.548
	Other/deformed vs. biconcave	0.35 (0.10–1.20)	0.095	0.130
	Age, per 10 years	0.82 (0.63–1.06)	0.126	—
	Female vs. male	0.59 (0.20–1.78)	0.350	—
Crepitus GEE	ADDwR vs. normal	1.40 (0.50–3.88)	0.519	0.548
	ADDwoR vs. normal	2.38 (1.00–5.67)	0.050	0.083
	Condylar flattening	0.86 (0.52–1.42)	0.548	0.548
	Age, per 10 years	1.32 (0.87–2.02)	0.194	—
	Female vs. male	0.70 (0.16–3.10)	0.643	—

OR, odds ratio; CI, confidence interval; GEE, generalized estimating equations. Both models used 216 joints nested in 108 patients with an exchangeable working correlation and robust standard errors. Clicking occurred in 63 joints and crepitus in 28. Age is per 10-year increase centered at 50 years, sex is female versus male, and MRI findings are presence versus absence. The three uncommon non-biconcave, non-flattened disc configurations were combined in the clicking model to reduce sparse-category instability. CIs are unadjusted; q values are Benjamini–Hochberg-adjusted across the 15 primary MRI predictor tests. The exploratory osseous LMO model was not included in this correction and is reported as exploratory. The crepitus findings warrant particular caution.

## Data Availability

The data presented in this study are available on request from the corresponding author.

## Supplementary Materials

The following supporting information can be downloaded at: https://www.mdpi.com/article/10.3390/jcm15218204/s1, Supplementary Material S1: Measurement and descriptive findings of inferior lateral pterygoid tendon thickness, including Table S1 and Figure S1 [29,30].

## Abbreviations

The following abbreviations are used in this manuscript:

ADDwoR	Anterior disc displacement without reduction
ADDwR	Anterior disc displacement with reduction
CI	Confidence interval
DC/TMD	Diagnostic Criteria for Temporomandibular Disorders
GEE	Generalized estimating equations
ICC	Intraclass correlation coefficient
IQR	Interquartile range
LMO	Limited mouth opening
MMO	Maximum mouth opening
MRI	Magnetic resonance imaging
OR	Odds ratio
SD	Standard deviation
SPSS	Statistical Package for the Social Sciences
TMD	Temporomandibular disorders
TMJ	Temporomandibular joint
VIF	Variance inflation factor

## References

[1] Tamimi D., Kocasarac H.D., Mardini S. (2019). Imaging of the Temporomandibular Joint. Semin. Roentgenol..

[2] Tanaka E., Detamore M.S., Mercuri L.G. (2008). Degenerative disorders of the temporomandibular joint: Etiology, diagnosis, and treatment. J. Dent. Res..

[3] de Oliveira L., Alves I.D.S., Vieira A.P.F., Passos U.L., Leite C.D.C., Gebrim E.S. (2023). Temporomandibular joint: From anatomy to internal derangement. Radiol. Bras..

[4] Cayon-Somacarrera S., Gutierrez-Rodriguez R., Munoz-Guerra M.F., Rodriguez-Campo F.J., Escorial-Hernandez V., Ocon-Alonso E.M. (2024). Unlocking the Temporomandibular Joint: CT, MRI, and Arthroscopic Correlation. Radiographics.

[5] Peck C.C., Goulet J.P., Lobbezoo F., Schiffman E.L., Alstergren P., Anderson G.C., de Leeuw R., Jensen R., Michelotti A., Ohrbach R. (2014). Expanding the taxonomy of the diagnostic criteria for temporomandibular disorders. J. Oral Rehabil..

[6] Whyte A., Boeddinghaus R., Bartley A., Vijeyaendra R. (2021). Imaging of the temporomandibular joint. Clin. Radiol..

[7] Matsubara R., Yanagi Y., Oki K., Hisatomi M., Santos K.C., Bamgbose B.O., Fujita M., Okada S., Minagi S., Asaumi J. (2018). Assessment of MRI findings and clinical symptoms in patients with temporomandibular joint disorders. Dentomaxillofac. Radiol..

[8] Vogl T.J., Lauer H.C., Lehnert T., Naguib N.N., Ottl P., Filmann N., Soekamto H., Nour-Eldin N.E.A. (2016). The value of MRI in patients with temporomandibular joint dysfunction: Correlation of MRI and clinical findings. Eur. J. Radiol..

[9] Topaloglu Yasan G., Adiloglu S., Tuz H.H., Sahar D. (2023). Evaluation of clinical signs and magnetic resonance imaging findings in patients with temporomandibular disorders. J. Craniomaxillofac. Surg..

[10] Sancar E., Yılmaz D., Parlak S., Bulut E.G., Ozer G., Hersek N.E. (2025). Exploring the relationship between clinical symptoms and MRI findings in temporomandibular joint disorders: A preliminary study. J. Oral Facial Pain Headache.

[11] Sang S., Ameli N., Almeida F.T., Friesen R. (2024). Association between clinical symptoms and MRI image findings in symptomatic temporomandibular joint (TMJ) disease: A systematic review. J. Craniomaxillofac. Surg..

[12] Schiffman E., Ohrbach R., Truelove E., Look J., Anderson G., Goulet J.P., List T., Svensson P., Gonzalez Y., Lobbezoo F. (2014). Diagnostic Criteria for Temporomandibular Disorders (DC/TMD) for Clinical and Research Applications: Recommendations of the International RDC/TMD Consortium Network and Orofacial Pain Special Interest Group. J. Oral Facial Pain Headache.

[13] Katzberg R.W., Tallents R.H. (2005). Normal and abnormal temporomandibular joint disc and posterior attachment as depicted by magnetic resonance imaging in symptomatic and asymptomatic subjects. J. Oral Maxillofac. Surg..

[14] Tomas X., Pomes J., Berenguer J., Quinto L., Nicolau C., Mercader J.M., Castro V. (2006). MR imaging of temporomandibular joint dysfunction: A pictorial review. Radiographics.

[15] Emshoff R., Rudisch A., Innerhofer K., Bosch R., Bertram S. (2001). Temporomandibular joint internal derangement type III: Relationship to magnetic resonance imaging findings of internal derangement and osteoarthrosis. An intraindividual approach. Int. J. Oral Maxillofac. Surg..

[16] Nozawa M., Ogi N., Ariji Y., Kise Y., Nakayama M., Nishiyama M., Naitoh M., Kurita K., Ariji E. (2020). Reliability of diagnostic imaging for degenerative diseases with osseous changes in the temporomandibular joint with special emphasis on subchondral cyst. Oral Radiol..

[17] Moss S.G., Schweitzer M.E., Jacobson J.A., Brossmann J., Lombardi J.V., Dellose S.M., Coralnick J.R., Standiford K.N., Resnick D. (1998). Hip joint fluid: Detection and distribution at MR imaging and US with cadaveric correlation. Radiology.

[18] Ohlmann B., Rammelsberg P., Henschel V., Kress B., Gabbert O., Schmitter M. (2006). Prediction of TMJ arthralgia according to clinical diagnosis and MRI findings. Int. J. Prosthodont..

[19] Larheim T.A., Westesson P., Sano T. (2001). Temporomandibular joint disk displacement: Comparison in asymptomatic volunteers and patients. Radiology.

[20] Katzberg R.W., Westesson P.L., Tallents R.H., Drake C.M. (1996). Anatomic disorders of the temporomandibular joint disc in asymptomatic subjects. J. Oral Maxillofac. Surg..

[21] Jeon K.J., Lee C., Choi Y.J., Han S.S. (2021). Analysis of three-dimensional imaging findings and clinical symptoms in patients with temporomandibular joint disorders. Quant. Imaging Med. Surg..

[22] Widmalm S.E., Westesson P.L., Brooks S.L., Hatala M.P., Paesani D. (1992). Temporomandibular joint sounds: Correlation to joint structure in fresh autopsy specimens. Am. J. Orthod. Dentofac. Orthop..

[23] Arayasantiparb R., Mitrirattanakul S., Kunasarapun P., Chutimataewin H., Netnoparat P., Sae-Heng W. (2020). Association of radiographic and clinical findings in patients with temporomandibular joints osseous alteration. Clin. Oral Investig..

[24] Manfredini D., Guarda-Nardini L. (2008). Agreement between Research Diagnostic Criteria for Temporomandibular Disorders and magnetic resonance diagnoses of temporomandibular disc displacement in a patient population. Int. J. Oral Maxillofac. Surg..

[25] Yatani H., Suzuki K., Kuboki T., Matsuka Y., Maekawa K., Yamashita A. (1998). The validity of clinical examination for diagnosing anterior disk displacement without reduction. Oral Surg. Oral Med. Oral Pathol. Oral Radiol. Endod..

[26] Sidebottom A.J. (2013). How do I manage restricted mouth opening secondary to problems with the temporomandibular joint?. Br. J. Oral Maxillofac. Surg..

[27] Palconet G., Ludlow J.B., Tyndall D.A., Lim P.F. (2012). Correlating cone beam CT results with temporomandibular joint pain of osteoarthritic origin. Dentomaxillofac. Radiol..

[28] Koh K.J., Park H.N., Kim K.A. (2013). Relationship between anterior disc displacement with/without reduction and effusion in temporomandibular disorder patients using magnetic resonance imaging. Imaging Sci. Dent..

[29] Litko M., Szkutnik J., Berger M., Różyło-Kalinowska I. (2016). Correlation between the lateral pterygoid muscle attachment type and temporomandibular joint disc position in magnetic resonance imaging. Dentomaxillofac. Radiol..

[30] Dergin G., Kilic C., Gozneli R., Yildirim D., Garip H., Moroglu S. (2012). Evaluating the correlation between the lateral pterygoid muscle attachment type and internal derangement of the temporomandibular joint with an emphasis on MR imaging findings. J. Craniomaxillofac. Surg..

