# Supplementary material for: Associations Between MRI Findings and Pain, Joint Sounds, and Limited Mouth Opening in Patients with Temporomandibular Disorders
Source: J Clin Med. 2026 Oct 22;15(21):8204. doi: 10.3390/jcm15218204 (PMC13764579; doi:10.3390/jcm15218204)
Supplement: Supplementary file 1 [file jcm-15-08204-s001.zip › jcm-4568469-supplementary.pdf]

# Supplementary Materials S1

## Measurement and descriptive findings of inferior lateral pterygoid tendon thickness

*Supplement to Associations between MRI findings and pain, joint sounds, and limited mouth opening in patients with temporomandibular disorders*

### S1 Rationale and scope

Inferior lateral pterygoid muscle (LPM) tendon thickness was assessed on magnetic resonance imaging (MRI) in 216 temporomandibular joints (TMJs) from 108 patients with temporomandibular disorders. The measurement was selected to characterize the condylar insertional region and describe its morphology across disc-position groups. Attachment-pattern studies examine LPM connections to the disc–condyle complex [1,2], whereas the present measure concerns the inferior tendon at the condylar neck. It does not classify superior-head attachment patterns. Tendon thickness was reported descriptively and was not included in the clinical outcome models.

### S2 Measurement protocol

Measurements were obtained on closed-mouth sagittal oblique proton-density images acquired with a 3 T scanner and a dedicated TMJ surface coil. In-plane spatial resolution was 0.5 × 0.5 mm, and section thickness was 3 mm. The inferior LPM tendon was identified at its insertion on the anterior aspect of the mandibular condylar neck, in the pterygoid fovea region. On the slice providing the clearest visualization, thickness was defined as the maximum perpendicular distance between the superior and inferior tendon margins at the insertional portion (Figure S1).

A radiologist with more than 10 years of experience in TMJ imaging performed the assessment while blinded to clinical findings. For intrareader assessment, the same radiologist repeated measurements in a random subset of 30 patients (60 bilateral TMJs) after an eight-week interval, blinded to the initial measurements and clinical data. Repeatability was evaluated using a two-way random-effects intraclass correlation coefficient (ICC) for absolute agreement of single measures.

### S3 Descriptive findings

Mean inferior LPM tendon thickness was 1.26 ± 0.48 mm across the 216 TMJs. Median thickness was descriptively greater in anterior disc displacement with reduction (ADDwR) and anterior disc displacement without reduction (ADDwoR) than in joints with normal disc position (Table S1). The intrareader ICC was 0.90 (95% confidence interval, 0.83–0.95).

Table S1. Inferior LPM tendon thickness by disc position.

| Disc position | Joints, n | Thickness, median (IQR), mm |
|---|---|---|
| Normal | 80 | 0.9 (0.7–1.15) |
| ADDwR | 79 | 1.3 (1.0–1.5) |
| ADDwoR | 57 | 1.5 (1.2–1.9) |

IQR, interquartile range. Values are joint-level descriptive summaries; no inferential comparisons of tendon thickness are reported. The normal-disc group comprised joints from symptomatic patients, rather than asymptomatic controls.

## S4 Measurement considerations

At the acquired in-plane resolution, the reported group medians spanned approximately two to three pixels. Partial-volume effects and selection of tendon boundaries may therefore influence measurement accuracy. The ICC documents repeatability of the measurement process and does not establish anatomical accuracy. The numerical differences between disc-position groups describe MRI measurements in this symptomatic sample; they do not establish tendon pathology or independent relationships with pain, joint sounds, or limited mouth opening.

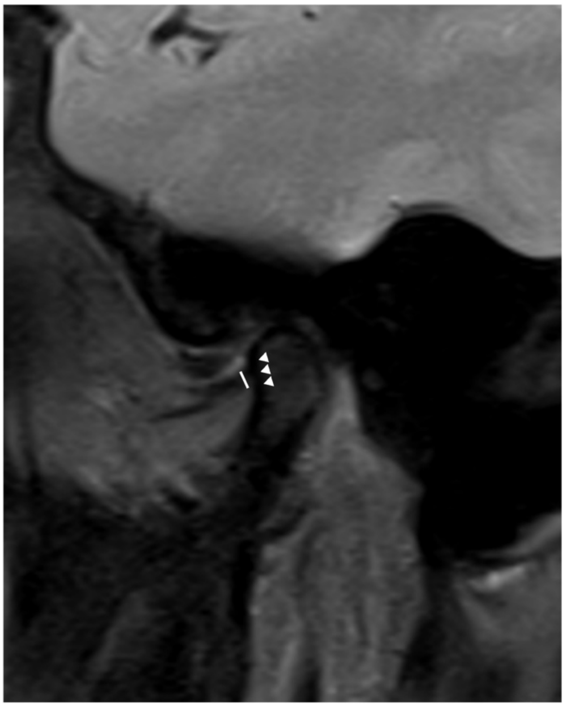

Figure S1. Measurement of inferior LPM tendon thickness. Closed-mouth sagittal oblique proton-density MR image showing the thickness measurement (white line) at the tendon insertion on the anterior aspect of the mandibular condylar neck (arrowheads).

## References


1. Litko, M.; Szkutnik, J.; Berger, M.; Różyło-Kalinowska, I. Correlation between the lateral pterygoid muscle attachment type and temporomandibular joint disc position in magnetic resonance imaging. *Dentomaxillofac. Radiol.* **2016**, *45*, 20160229. https://doi.org/10.1259/dmfr.20160229.

2. Dergin, G.; Kilic, C.; Gozneli, R.; Yildirim, D.; Garip, H.; Moroglu, S. Evaluating the correlation between the lateral pterygoid muscle attachment type and internal derangement of the temporomandibular joint with an emphasis on MR imaging findings. *J. Craniomaxillofac. Surg.* **2012**, *40*, 459–463. https://doi.org/10.1016/j.jcms.2011.08.002.
